# Heart Failure Prevalence Rates and Its Association with Other Cardiovascular Diseases and Chronic Kidney Disease: SIMETAP-HF Study

**DOI:** 10.3390/jcm12154924

**Published:** 2023-07-26

**Authors:** Antonio Ruiz-García, Adalberto Serrano-Cumplido, Carlos Escobar-Cervantes, Ezequiel Arranz-Martínez, Miguel Turégano-Yedro, Vicente Pallarés-Carratalá

**Affiliations:** 1 Pinto Primary Care Center, Lipids and Cardiovascular Prevention Unit, University Health Centre, 28320 Madrid, Spain; antoniodoctor@gmail.com; 2 Department of Medicine, European University of Madrid, 28005 Madrid, Spain; 3 Repelega Primary Care Center, 48920 Bizkaia, Spain; adal1953@hotmail.com; 4 Department of Cardiology, La Paz University Hospital, 28046 Madrid, Spain; carlos.escobar@salud.madrid.org; 5 San Blas Health Centre, 28981 Madrid, Spain; ezequielarranz@gmail.com; 6 Casar de Cáceres Health Centre, 10190 Caceres, Spain; tureyedro@gmail.com; 7 Health Surveillance Unit, Mutual Insurance Union, 12004 Castellon, Spain; 8 Department of Medicine, Jaume I University, 12006 Castellon, Spain

**Keywords:** heart failure, atherosclerotic cardiovascular disease, cardiovascular risk factors, prevalence, adults

## Abstract

Introduction and objectives: Heart failure (HF) is a major health problem that causes high mortality and hospitalization rates. This study aims to determine the HF prevalence rates in populations aged both ≥18 years and ≥50 years and to assess its association with cardiovascular diseases and chronic kidney disease. Methods: A cross-sectional observational study was conducted in a primary care setting, with a population-based random sample of 6588 people aged 18.0–102.8 years. Crude and adjusted prevalence rates of HF were calculated. The associations of renal and cardiometabolic factors with HF were assessed in both populations using univariate, bivariate and multivariate analysis. Results: The HF crude prevalence rates were 2.8% (95%CI: 2.4–3.2) in adults (≥18 years), and 4.6% (95%CI: 4.0–5.3) in the population aged ≥ 50 years, without significant differences between males and females in both populations. The age- and sex-adjusted prevalence rates were 2.1% (male: 1.9%; female: 2.3%) in the overall adult population, and 4.5% (male: 4.2%; female: 4.8%) in the population aged ≥ 50 years, reaching 10.0% in the population aged ≥ 70 years. Atrial fibrillation, hypertension, low estimated glomerular filtration rate (eGFR), coronary heart disease (CHD), stroke, sedentary lifestyle, and diabetes were independently associated with HF in both populations. A total of 95.7% (95%CI: 92.7–98.6) of the population with HF had an elevated cardiovascular risk. Conclusions: This study reports that HF prevalence increases from 4.5% in the population over 50 years to 10% in the population over 70 years. The main clinical conditions that are HF-related are sedentary lifestyle, atrial fibrillation, hypertension, diabetes, low eGFR, stroke, and CHD.

## 1. Introduction

Heart failure (HF) is a complex clinical syndrome that results from any structural and/or functional cardiac disturbances. Any cardiac pathology such as myocardial dysfunction (systolic and/or diastolic), heart valve disease, pericarditis, endocarditis, cardiac rhythm and conduction disturbances can cause or contribute to HF [1,2,3].

HF is usually preceded by diseases or conditions which, in turn, may be the cause of other alterations [1,2,3,4] (Figure S1 Supplementary Materials). The most prevalent causes of HF have changed over time [4,5] and are probably different depending on the economic level of different countries [6]. In developed countries, the main causes associated with HF are coronary heart disease (CHD) and arterial hypertension (HTN) [1,2,3,4,5,6].

On the other hand, patients with HF tend to accumulate other comorbidities (CHD, atrial fibrillation [AF], atrial flutter, peripheral arterial disease, stroke, HTN, anemia, obesity, hypercholesterolemia, diabetes mellitus [DM], rheumatoid arthritis, chronic obstructive pulmonary disease and chronic kidney disease [CKD]), either individually or grouped (mean 3.9 comorbidities per patient, with a range of 0 to 9), and this is more frequent in patients with HF with preserved ejection fraction compared to those with reduced ejection fraction [6]. The presence and control of these comorbidities influence the results and quality of life of patients with HF.

HF diagnosis carries an increased risk of morbidity and mortality. HF is a well-recognized public health problem worldwide that causes an increasing health and economic burden due to numerous hospital admissions and high mortality [7,8]. The age-adjusted incidence of HF is decreasing in developed countries, possibly due to better therapeutic management of the disease, although its overall incidence is increasing due to population ageing [7,8,9,10,11,12].

HF prevalence rates range from 1% to 2% of the adult population. Prevalence increases with age, from less than 1% for those younger than 55 years to >10% in those older than 70 years [8,9,10,11,12,13,14,15,16]. It is likely that the true prevalence of HF is higher because patients with HF are not recognized or diagnosed in a primary care setting, especially those with preserved ejection fraction [17].

A SIMETAP-HF study was designed to determine the crude and adjusted prevalence rates of HF both in the adult population (≥18 years) and in the population older than 50 years and to assess its association with other cardiovascular diseases and CKD.

## 2. Methods

SIIMETAP-HF is a sub-study of the SIMETAP study [18], a multicenter cross-sectional observational study, authorized by the Health Service of the Community of Madrid (SERMAS according to its initials in Spanish), which 121 physicians participated in. The physicians were selected competitively to reach the necessary sample size and belonged to 64 primary care centers (25.6% from de SERMAS healthcare centers). Simple random sampling of 5.45% of the target population aged 18 years and over (194,073 adults) assigned to GPs was performed using random numbers drawn from the Excel function RAND.BETWEEN (bottom, top). Terminally ill patients or those with cognitive impairments, institutionalized persons, and pregnant women were excluded as per protocol. After a response rate of 62.9%, 6588 study subjects were selected with informed consent and with the necessary clinical and laboratory data to be evaluated. The researchers entered study data from January to December 2015, and collected data based on the most recent biochemical parameters determined from blood and urine tests taken during the previous year. For the purposes of this study, registry of HF diagnosis (International Classification of Diseases, Tenth Revision, Clinical Modification [ICD-10-CM] code: I50; International Classification of Primary Care, 2nd edition [ICPC-2] code: K77) in the patient’s medical record was considered HF, without differentiating by phenotypes based on the measurement of the left ventricular ejection fraction or based on the severity of symptoms and physical activity. The concepts and criteria of the assessed clinical conditions and variables are shown in Table S1 (Supplementary Materials). The study was approved by the Research Commission Deputy Management of Planning and Quality Primary Care—Autonomous Community of Madrid Primary Care Management on 8 November 2010 (Approval Code: 05/2010).

Qualitative variables were analyzed using percentages, a chi-square test, and odds ratios, with a 95% confidence interval (CI). The Shapiro–Wilk test was used to check the data fitting to normal distribution for quantitative variables. If the variables showed normal distribution, they were analyzed using the arithmetic mean, standard deviation (SD) and Student’s *t*-test or analysis of variance. The median and interquartile range (IQR) of age were determined. Prevalence rates were determined in populations aged ≥18, ≥50, ≥60, and ≥70 years of age. The age- and sex-adjusted prevalence rates were calculated by the direct method, using standardized ten-year age groups of the Spanish population as of January 2015 according to the National Institute of Statistics [19].

Bivariate and multivariate analyses were performed in populations aged ≥18 years and ≥50 years. To assess the individual effect of comorbidities and cardiovascular risk factors (CVRFs) on the dependent variable HF, multivariate logistic regression analysis was performed using the backward stepwise method. Initially, all the variables that showed association in the univariate analysis up to a *p*-value of <0.10 were introduced into the model, except for erectile dysfunction because it affects only men and CUN-BAE-obesity [20] and metabolic syndrome (MetS) [21] because both variables integrate some factors or criteria assessed independently in the analysis (Table S1, Supplementary Materials). Subsequently, the variable that contributed least to the fit of the analysis was eliminated at each step. All tests were considered statistically significant if the two-tailed *p*-value was <0.05. The Statistical Package for the Social Sciences was used for the statistical analysis.

## 3. Results

The study population consisted of 6588 adults aged 18.0–102.8, whose mean (SD) age was 55.1 (17.5) years, and median (IQR) was 54.7 (41.7–68.1) years. The difference in percentage between males (44.1% [95%CI: 42.9–45.3%]) and females (55.9% [95%CI: 54.7–57.1%]) was significant (*p* < 0.001). The median (IQR) ages of the male and female populations were 55.0 (42.4–67.5) years and 54.5 (41.0–68.8) years, respectively, with the difference in mean (SD) age between males (55.3 [16.9] years) and females (55.0 [18.0] years) being non-significant (*p* = 0.634).

### 3.1. HF Prevalence Rates

**Figure 1:**
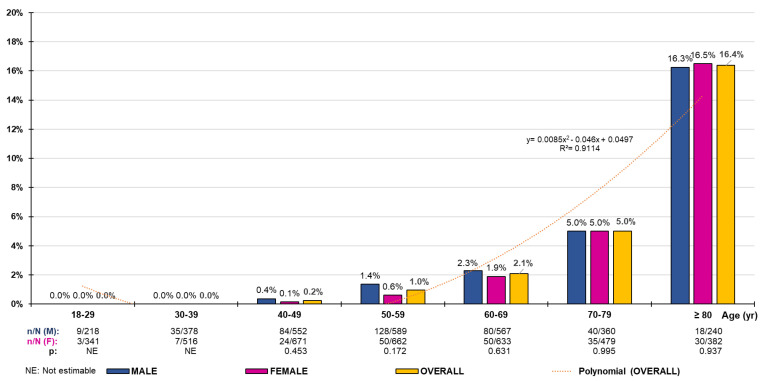
Heart failure prevalence rates by age group. n: number of cases; N: sample size; M: male; F: female; p: *p*-value of the difference in percentages (M–F).

The crude and adjusted prevalence rates of HF in populations aged ≥18, ≥50, ≥60, and ≥70 years are shown in Table 1. The differences in prevalence rates between males and females were non-significant both globally (≥18 years) and by age group (Figure 1). The distribution of HF prevalence rates by ten-year age group increased according to the polynomial function y = 0.0085x^2^ − 0.046x + 0.0497 (R^2^ = 0.911). The prevalence rates were anecdotal in the population aged <50 years (Figure 1).

### 3.2. Analysis of Populations ≥18 Years with and without HF

The median (IQR) ages of the populations with and without HF were 80.9 (72.0–88.7) years and 54.3 (41.4–67.2) years, respectively, with the difference in mean (SD) ages (23.9 [95%CI: 21.4–26.4] years) between both populations being significant (*p* < 0.001) (Table 2). The difference in the female percentage (0.6% [95%CI: −6.7–7.9]) between the populations with and without HF was non-significant (*p* = 0.868) (Table 3). In the population with HF, the difference in mean (SD) age between males (78.8 [10.2] years) and females (76.4 [12.2] years) was significant (*p* = 0.040).

All quantitative clinical variables were significantly higher in the population with HF than in the population without HF, except for diastolic blood pressure, total cholesterol (TC), high-density lipoprotein cholesterol (HDL-C), non-HDL-C, low-density lipoprotein cholesterol (LDL-C), non-HDL-C/HDL-C, ALT, and estimated glomerular filtration rate (eGFR), which were significantly higher in the population without HF. The differences in triglycerides (TG), very-low-density lipoprotein cholesterol (VLDL-C), TG/HDL-C, and AST were non-significant (Table 2).

All the ORs of the CVRFs and comorbidities showed a significant association with HF, except for overweight and prediabetes, whose differences were non-significant. The ORs for smoking, alcoholism, and low, moderate, and high cardiovascular risk (CVR) showed a significant association with the population without HF. A total of 95.7% [95%CI: 92.7–98.6]) of the population with HF had a high or very high CVR according to SCORE [22] and SCORE-OP [23] (Table 3). The multivariate analysis showed that AF, HTN, low eGFR, CHD, stroke, low HDL-C, sedentary lifestyle, and DM were independently associated with HF (Table 4).

### 3.3. Analysis of Populations ≥50 Years with and without HF

The median (IQR) ages of the populations with and without HF were 81.1 (73.3–86.7) years and 65.2 (57.5–74.6) years, respectively, with the difference in mean (SD) ages (12.4 [95%CI: 10.8–14.0] years) between both populations being significant (*p* < 0.001) (Table 2). The difference in the female percentage (1.9% [95%CI: 5.5–9.3]) between the populations with and without HF was non-significant (*p* = 0.619) (Table 3). In the population with HF, the difference in mean (SD) age between males (80.2 [9.6] years) and females (77.2 [11.4] years) was close to statistical significance (*p* = 0.057).

All quantitative clinical variables were significantly higher in the population with HF than in the population without HF, except for diastolic blood pressure, TC, HDL-C, non-HDL-C, LDL-C, non-HDL-C/HDL-C, and eGFR, which were significantly higher in the population without HF. The differences for systolic blood pressure, TG, VLDL-C, TG/HDL-C, triglyceride-glucose (TyG) index, and AST were non-significant (Table 2).

All the ORs of the CVRFs and comorbidities showed a significant association with HF, except for overweight, abdominal obesity, increased WHtR, prediabetes, hypercholesterolaemia, hypertriglyceridaemia, and atherogenic dyslipidemia, whose differences were non-significant. The ORs for smoking, alcoholism and low, moderate and high CVR showed a significant association with the population without HF. A total of 97.2% [95%CI: 94.8–99.6] of the population with HF had a high or very high CVR according to SCORE [22] and SCORE-OP [23] (Table 3). The multivariate analysis showed that the same variables independently associated with HF in the overall adult population were also associated in the population aged ≥50 years (Table 4). The main results are summarized in the Graphical Abstract.

## 4. Discussion

### 4.1. HF Prevalence Rates

The healthcare burden of HF is increasing worldwide, probably due to population ageing and lower mortality because of better management of the disease and its associated factors [8,9,10,11,12,13]. Consequently, the use of healthcare resources and hospitalizations readmissions, and outpatient visits due to HF have been increasing [16,24,25,26]. Seferovic et al. [26] showed that the incidence of HF in Spain was 2.76 per 1000 person-years and the prevalence was 12.0 per 1000 persons. The adjusted prevalence rates of HF in the present study increased with age (R^2^ = 0.911), from 2.1% in the population aged ≥18 years to 10.0% in the population aged ≥70 years, and was slightly higher in females in all age groups. HF cases are concentrated in the older age groups and are anecdotal in those under 50 years of age (Figure 1). It should be noted that the mean age of the subjects with HF in the SIMETAP-HF study (78.4 years) is similar to the mean age found in Störk et al.’s study [27] (76.2 years). The prevalence rates in the PRICE study [28] ranged from 1.3% in the 45–54 age group to 16.1% in those older than 74 years. HF prevalence in the population over 50 years was 4.5%, whereas the PRICE study [28] showed a prevalence of 6.8% in the population over 45 years. We observed that women with HF were younger than men, both in the ≥50 years group (mean 77.2 years [female] vs. 80.2 [male]) and in the ≥18 years group (76.4 years [female] vs. 78.8 [male]), which is probably due to gender differences in terms of predominant etiology (CHD in men vs. HTN and DM in women) [29].

### 4.2. Clinical Conditions and Factors Associated with HF

In a review by Khan et al. [30], although the prevalence of smoking in patients with HF has been decreasing in recent years, hyperlipidemia, CHD, DM, HTN, CKD and AF have increased over time. The SIMETAP-HF study found that 22 comorbidities were more frequent in the population aged ≥18 years with HF than in non-HF, and they were reduced to 15 when the analysis focused on the population aged ≥50 years (Table 3). Loosen et al.’s study [31] showed that 36 previously defined comorbidities were more frequent in HF patients, but regression analysis showed that only 19 of them were significantly associated with it. The multifactorial analysis performed in SIMETAP-HF showed that sedentary lifestyle, HTN, DM AF, CHD, stroke, and low eGFR were the factors independently associated with HF (Table 4).

Physical inactivity has been associated with an increased risk of HF worsening and all-cause mortality in patients with HF [32]. In our study, 64% of patients with HF had a sedentary lifestyle, and this was independently associated with HF (OR 2.0).

There is a correlation between obesity and adiposity, especially abdominal fat, with cardiovascular disease [33]. The obesity and abdominal obesity prevalence rates were higher in patients with HF (41% and 61%, respectively) than in those without HF (27% and 44%, respectively). A study conducted in China showed that the highest WHtR values (≥0.5) constituted an independent risk factor for all-cause mortality, cardiovascular mortality and HF rehospitalization [34]. In patients with HF in the present study, the prevalence of elevated WHtR was 76%. Adiposity was also highly prevalent among HF patients in our study. The Jackson Heart study showed that visceral adiposity was associated with hospitalization for incident HF, pericardial adiposity and mortality [35].

HTN is the clinical condition most frequently associated with HF. A systematic review of HTN trials concluded that 28.9% of hypertensive patients had developed HF [36]. We found that more than 90% of patients with HF had HTN. Pulse pressure has also been linked to CVR [37]. Pulse pressure in the present study was higher in subjects with HF than in those without HF. Hypercholesterolemia and low HDL-C levels were also more common in HF patients. High levels of non-HDL cholesterol or low HDL-C levels are associated with the HF incidence [38].

Abnormal glucose regulation and DM maintain a close and bidirectional relationship with HF, and the presence of DM increases mortality and hospitalizations in patients with HF [39]. Up to 27% and 42% of patients with HF in the SIMETAP-HF study had prediabetes and DM, respectively. Insulin resistance is the link between DM and MetS. In our study, the MetS prevalence in patients with HF (82%) was similar to that shown in other studies [40].

**Figure 2:**
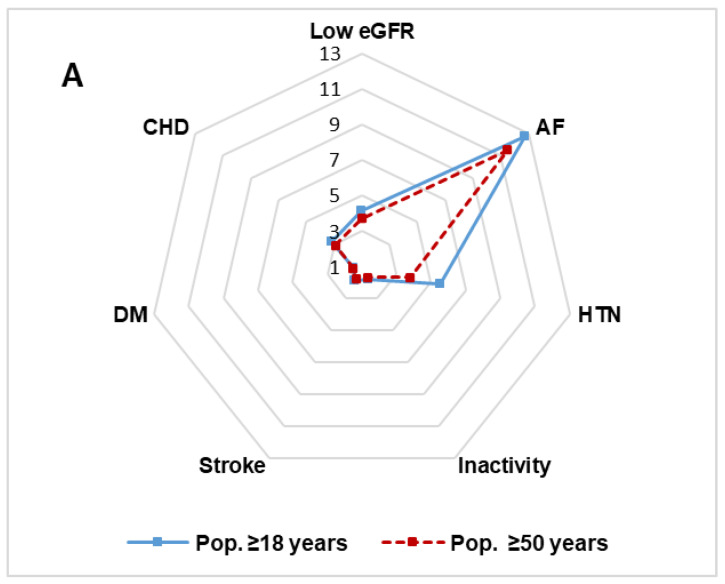

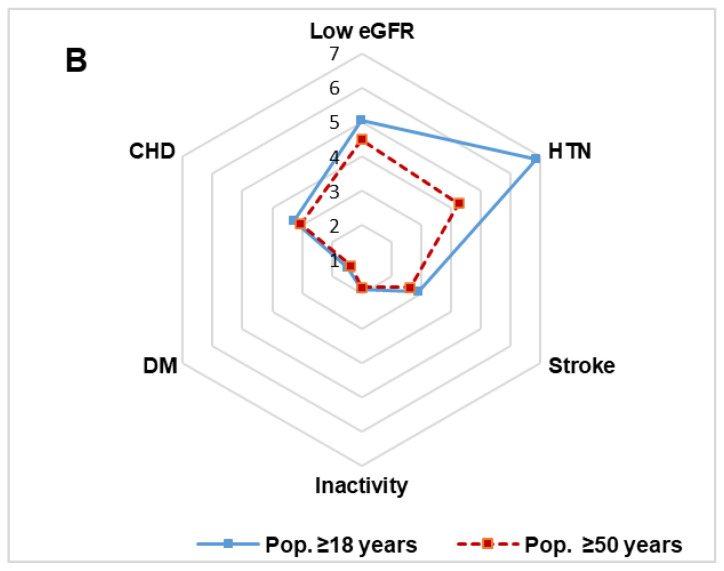
Radar chart of clinical conditions and comorbidities independently associated with heart failure. (**A**). Multivariate analysis of factors associated with HF. (**B**). Multivariate analysis of factors associated with HF excluding AF. Numbers: odds ratio; AF: atrial fibrillation; CHD: coronary heart disease. DM: diabetes mellitus; HTN: hypertension; Inactivity: sedentary lifestyle; Low eGFR: estimated glomerular filtration rate <60 mL/min/1.73 m^2^.

AF is strongly associated with an increased risk of HF as these closely related pathologies that often coexist predispose to each other and share risk factors such as HTN, DM, CHD, and valve disease [41,42,43]. The present study shows a strong association of AF with HF (OR 11.5) due to the higher prevalence of AF in patients with HF (46.4%) than the Loosen et al., study [31] (18.6%). In order to better differentiate between the associations of the main clinical conditions or comorbidities with HF, Figure 2A,B show the results of multivariate analysis, including or not including AF, respectively. AF prevalence in the subjects with HF in our study (46%) is intermediate compared to that of other studies [32,42,43]. The differences are probably related to the study design, the age groups analyzed, the control of CVRFs associated with HF, and the greater or lesser availability of echocardiography or natriuretic peptides to diagnose HF in suspected cases.

Erectile dysfunction is an early predictor of cardiovascular events and HF, and HF itself can worsen sexual health due to its comorbidities. In our study, 71% of patients with HF had erectile dysfunction, similar to the other studies [44] (80%). The heart and kidneys act synergistically to maintain blood pressure and homeostasis such that any alteration of one of them leads to the deterioration of the other [45,46]. In the present study, the presence of low eGFR (48%) and CKD (55%) in patients with HF is in line with findings in the Löfman et al., study [47], in which 51% of FH patients had a low eGFR.

CHD is the main etiological factor in patients with HF [46,48]. The German EuroAspire IV cohort study [49] showed that 44.2% of patients with CHD had HF. Our study reports that about 30% of patients with HF had CHD, which is similar to the Swedish population and higher than the Chinese population [50]. The study by Loosen et al. [31] reported a lower association with HF (HR 1.5) probably because the median age of the population without HF (76 years) was much higher than that of our study (54.3 years).

The high prevalence of all these conditions, the metabolic, renal and cardiovascular factors suffered by patients with HF, and the mean age (78.4 years) justifies that more than 85% of the subjects have a very high CVR.

### 4.3. Strengths and Limitations

The main limitations of this study were the inability to determine causality or to estimate incidence rates, inter-interviewer variability, possible under-reporting in the medical records of the HF diagnosis, possible heterogeneity of the measurement and laboratory equipment, and HF underdiagnoses due to the per protocol exclusion of pregnant women, the terminally ill, and institutionalized or cognitively impaired patients. The main strengths include the population-based random selection, a large sample with people aged 18.0–102.8 years, the determination of both crude and adjusted prevalence rates of HF, and the assessment of the possible association of HF with numerous cardiometabolic and renal variables.

### 4.4. Clinical Implications

The high prevalence of HF in the population over 50 years of age entails serious socioeconomic and health consequences due to the increase in hospitalizations due to HF and cardiovascular mortality. Assessing the epidemiological magnitude of HF is essential to better plan prevention policies aimed at reducing the high healthcare and economic burden that it causes, both in primary care and in a hospital setting, to optimize available health resources and to improve medical care and quality of life for patients. HF is strongly influenced by age after 50 years, so its prevalence rates should always be age-adjusted to compare them with other populations. We hope that this study improves knowledge of HF prevalence and that it contributes to understanding the importance and magnitude of CVRFs and comorbidities associated with HF.

## 5. Conclusions

HF is a complex syndrome that constitutes a serious health problem worldwide, closely related to the aging of the population, which accumulates a constellation of comorbidities that accelerate its progression and increase the risk of mortality and hospitalization.

The assessment of the current prevalence of HF among the adult population and its association with ASCVD and CKD is crucial because it can help in the rational management of patients with HF. This study investigated the crude and adjusted prevalence rates of HF and its comorbid conditions. It confirmed the close relationship between age and the prevalence of HF, without differences by sex, being 2.1% in the adult population, 4.6% in those ≥50 years, and reaching 10% in those ≥70 years. The confluence of all the comorbidities independently associated with HF and the patients’ ageing determined that their CVR was very high.

The early detection of HF-related comorbidities will allow the comprehensive management of HF, which would include healthy lifestyle modifications, and an approach to obesity, HTN, and DM, which would delay the worsening of HF and the development of AF, CKD, and ASCVD.

## 6. Key Points

### 6.1. What Is Known about the Topic?

- Heart failure prevalence rates vary according to the income and development of countries.
- Although heart failure mortality is stabilizing over time, heart failure hospitalization is increasing due to population ageing, which carries a high economic and healthcare burden.
- There are many cardiometabolic and renal factors associated with heart failure.

### 6.2. What Does This Study Add?

- Heart failure is strongly influenced by age after 50 years.
- Heart failure prevalence rates increase from 2.1% in the overall adult population to 10% in people over 70 years.
- Physicians should be aware that atrial fibrillation, hypertension, diabetes, coronary heart disease, stroke, low estimated glomerular filtration rate and sedentary lifestyle are clinical conditions independently associated with heart failure.
- Knowing the cardiometabolic and renal factors associated with heart failure would facilitate its early detection and management to reduce its severity and hospitalization rates.

## Figures and Tables

**Table 1:** Heart failure prevalence rates.

Age Groups (Years)	Overall Prevalence % (95%CI)	Crude Prevalence (M) % (95%CI)	Crude Prevalence (F) % (95%CI)	*p*	Overall Adjusted Prevalence (%)	Adjusted Prevalence (M) (%)	Adjusted Prevalence (F) (%)
≥18	2.79 (2.40–3.19)	2.75 (2.16–3.35)	2.82 (2.29–3.36)	0.864	2.11	1.90	2.32
≥50	4.63 (3.97–5.29)	4.44 (3.48–5.41)	4.78 (3.88–5.68)	0.615	4.49	4.19	4.78
≥60	6.35 (5.42–7.28)	6.00 (4.64–7.36)	6.63 (5.37–7.89)	0.509	6.53	6.00	6.95
≥70	9.86 (8.33–11.38)	9.50 (7.15–11.85)	10.10 (8.09–12.12)	0.705	10.00	9.37	10.44

CI: confidence interval; M: male; F: female; p: *p*-value of difference in percentages (M–F).

**Table 2:** Clinical characteristics of populations with and without heart failure.

	Population ≥ 18 Years					Population ≥ 50 Years				
	With Heart Failure		Without Heart Failure		Difference in Means	With Heart Failure		Without Heart Failure		Difference in Means
	N	Mean (SD)	N	Mean (SD)	*p*-Value	N	Mean (SD)	N	Mean (SD)	*p*-Value
Age (years)	184	78.4 (11.2)	6404	54.5 (17.2)	<0.001	181	78.9 (10.5)	3731	66.5 (10.8)	<0.001
Body mass index (kg/m^2^)	184	29.8 (6.3)	6404	27.4 (5.1)	<0.001	181	29.8 (6.3)	3731	28.5 (4.8)	0.001
Abdominal circumference (cm)	184	99.5 (14.7)	6404	93.2 (14.0)	<0.001	181	99.6 (14.7)	3731	96.7 (13.0)	0.004
Waist-to-height ratio	184	0.63 (0.10)	6404	0.57 (0.09)	<0.001	181	0.63 (0.10)	3731	0.60 (0.08)	<0.001
Adiposity (%)	184	39.8 (8.6)	6404	34.9 (8.6)	<0.001	181	40.0 (8.5)	3731	37.3 (7.8)	<0.001
Systolic blood pressure (mmHg)	184	127.7 (16.4)	6404	121.8 (15.4)	<0.001	181	127.5 (16.2)	3731	126.5 (14.7)	0.354
Diastolic blood pressure (mmHg)	184	71.4 (9.9)	6404	73.4 (9.8)	0.006	181	71.2 (9.8)	3731	75.0 (9.2)	<0.001
Pulse pressure (mmHg)	184	56.3 (14.0)	6404	48.4 (11.8)	<0.001	181	56.3 (13.9)	3731	51.4 (12.4)	<0.001
Fasting plasma glucose (mg/dL) ^a^	184	107.6 (32.1)	6404	95.7 (25.7)	<0.001	181	108.0 (32.2)	3731	101.8 (28.2)	0.004
Glycated haemoglobin A1c (%) ^b^	166	6.15 (1.03)	5067	5.62 (0.89)	<0.001	164	6.15 (0.95)	3115	5.85 (0.95)	<0.001
Total cholesterol (mg/dL) ^c^	184	171.9 (42.8)	6404	193.4 (39.1)	<0.001	181	171.3 (41.6)	3731	197.5 (38.7)	<0.001
HDL-C (mg/dL) ^c^	184	52.0 (15.4)	6404	54.9 (14.7)	0.007	181	52.1 (15.5)	3731	55.0 (14.9)	0.009
LDL-C (mg/dL) ^c^	183	95.4 (36.3)	6343	114.7 (34.3)	<0.001	180	94.6 (35.7)	3699	117.3 (34.3)	<0.001
VLDL-C (mg/dL) ^c^	183	24.3 (12.1)	6343	22.9 (12.3)	0.136	180	24.4 (12.2)	3699	24.4 (12.2)	0.987
Non-HDL-C (mg/dL) ^c^	184	120.0 (40.8)	6404	138.5 (38.2)	<0.001	181	119.3 (40.6)	3731	142.4 (36.8)	<0.001
Triglycerides (mg/dL) ^d^	184	123.8 (64.2)	6404	120.4 (83.7)	0.590	181	124.4 (64.6)	3731	127.0 (75.8)	0.650
Non-HDL-C/HDL-C	184	2.51 (1.16)	6404	2.73 (1.13)	0.011	181	2.49 (1.16)	3731	2.78 (1.07)	<0.001
Triglycerides/HDL-C	184	2.73 (1.88)	6404	2.52 (2.57)	0.289	181	2.74 (1.89)	3731	2.64 (2.27)	0.576
TyG index	184	8.65 (0.62)	6404	8.49 (0.60)	<0.001	181	8.66 (0.62)	3731	8.62 (0.57)	0.439
Uric acid (mg/dL) ^e^	175	5.78 (1.90)	5993	4.94 (1.47)	<0.001	172	5.78 (1.90)	3522	5.13 (1.47)	<0.001
Aspartate aminotransferase (U/L)	138	21.6 (10.1)	4683	23.1 (43.7)	0.685	136	21.7 (10.1)	2716	23.7 (41.8)	0.285
Alanine aminotransferase (U/L)	180	21.7 (10.7)	6242	25.0 (17.1)	0.011	177	21.7 (10.7)	3630	25.0 (16.0)	0.007
Gamma-glutamyl transferase (U/L)	173	46.7 (51.7)	5935	33.1 (50.7)	<0.001	171	47.0 (51.9)	3453	36.2 (45.2)	0.002
Creatinine (mg/dL) ^f^	184	1.10 (0.50)	6404	0.84 (0.28)	<0.001	181	1.10 (0.50)	3731	0.86 (0.30)	<0.001
eGFR (mL/min/1.73 m^2^)	184	62.0 (20.5)	6404	91.4 (19.9)	<0.001	181	61.5 (20.1)	3731	82.3 (17.4)	<0.001
ACR (mg/g) ^g^	184	44.2 (95.8)	6404	15.6 (58.9)	<0.001	181	44.8 (96.5)	3731	19.7 (71.6)	<0.001

ACR: urine albumin-creatinine ratio; eGFR: estimated glomerular filtration rate; HDL-C: high-density lipoprotein cholesterol; LDL-C: low-density lipoprotein cholesterol; non-HDL-C: non-high-density lipoprotein cholesterol; TyG index: triglyceride and glucose index; VLDL-C: very-low-density lipoprotein cholesterol. The definitions of the variables or clinical conditions are shown in Table S1 (Supplementary Materials). ^a^ To convert from mg/dL to mmol/L, multiply by 0.05556. ^b^ To convert from % (DCCT) to mmol/mol (IFCC), multiply by 0.09148 and add 2.152. ^c^ To convert from mg/dL to mmol/L, multiply by 0.02586. ^d^ To convert from mg/dL to mmol/L, multiply by 0.01129. ^e^ To convert from mg/dL to mmol/L, multiply by 0.05948. ^f^ To convert from mg/dL to mmol/L, multiply by 0.08842. ^g^ To convert from mg/g to mg/mmol, multiply by 0.01131.

**Table 3:** Risk factors and comorbidities in populations with and without heart failure.

	Population ≥ 18 Years				Population ≥ 50 Years			
	With HF N = 184	Without HF N = 6404	OR	*p*-Value	With HF N = 181	Without HF N = 3731	OR	*p*-Value
Male	80 (43.5)	2824 (44.1)	1.0 (0.7–1.3)	0.867	78 (43.1)	1678 (45.0)	0.9 (0.7–1.3)	0.619
Current smoking	13 (7.1)	1413 (22.1)	3.7 (2.1–6.6)	<0.001	11 (6.1)	654 (17.5)	0.3 (0.2–0.6)	<0.001
Alcoholism	8 (4.3)	602 (9.4)	2.3 (1.1–4.7)	0.020	6 (3.3)	351 (9.4)	0.3 (0.5–0.8)	0.005
Sedentary lifestyle	119 (64.7)	2960 (46.2)	2.1 (1.6–2.9)	<0.001	116 (64.1)	1761 (47.2)	2.0 (1.5–2.7)	<0.001
Overweight	67 (36.4)	2449 (38.2)	0.9 (0.7–1.3)	0.615	65 (35.9)	1595 (42.7)	0.8 (0.6–1.0)	0.069
Obesity	76 (41.3)	1757 (27.4)	1.9 (1.4–2.5)	<0.001	76 (42.0)	1274 (34.1)	1.4 (1.0–1.9)	0.030
Abdominal obesity	113 (61.4)	2809 (43.9)	2.0 (1.5–2.8)	<0.001	112 (61.9)	2051 (55.0)	1.3 (1.0–1.8)	0.068
Adiposity	175 (95.1)	4657 (72.7)	7.3 (3.7–14.3)	<0.001	173 (95.6)	3375 (90.5)	2.3 (1.1–4.7)	0.021
High WHtR	138 (75.0)	3558 (55.6)	2.4 (1.7–3.4)	<0.001	138 (76.2)	2649 (71.0)	1.3 (0.9–1.9)	0.128
Prediabetes	49 (26.6)	1400 (21.9)	1.3 (0.9–1.8)	0.124	48 (26.5)	1065 (28.5)	0.9 (0.6–1.3)	0.555
Diabetes	76 (41.3)	959 (15.0)	4.0 (3.0–5.4)	<0.001	76 (42.0)	860 (23.1)	2.4 (1.8–3.3)	<0.001
Hypertension	166 (90.2)	2381 (37.2)	15.6 (9.6–25.4)	<0.001	165 (91.2)	2115 (56.7)	7.9 (4.7–13.2)	<0.001
Hypercholesterolaemia	146 (79.3)	3955 (61.8)	2.4 (1.7–3.4)	<0.001	144 (79.6)	2886 (77.4)	1.1 (0.8–1.4)	0.488
Low HDL-C	74 (40.2)	745 (27.2)	1.8 (1.3–2.4)	<0.001	73 (40.3)	1076 (28.8)	1.7 (1.2–2.3)	0.001
Hypertriglyceridaemia	71 (38.6)	1876 (29.3)	1.5 (1.1–2.1)	0.006	71 (39.2)	1320 (35.4)	1.2 (0.9–1.6)	0.291
Atherogenic dyslipidemia	40 (21.7)	901 (14.1)	1.7 (1.2–2.4)	0.003	40 (22.1)	637 (17.1)	1.4 (1.0–2.0)	0.081
Hyperuricemia	49 (27.8)	734 (12.2)	2.8 (2.0–3.9)	<0.001	48 (27.7)	535 (15.2)	2.1 (1.5–3.0)	<0.001
Hepatic steatosis	31 (16.8)	562 (8.8)	2.1 (1.4–3.1)	<0.001	31 (17.1)	400 (10.7)	1.7 (1.2–2.6)	0.007
Metabolic syndrome	151 (82.1)	2700 (42.2)	6.3 (4.3–9.2)	<0.001	150 (82.9)	2252 (60.4)	3.2 (2.1–4.7)	<0.001
Coronary heart disease	55 (29.9)	266 (4.2)	9.8 (7.0–13.8)	<0.001	54 (29.8)	248 (6.6)	6.0 (4.2–8.4)	<0.001
Stroke	40 (21.7)	210 (3.3)	8.2 (5.6–11.9)	<0.001	39 (21.5)	194 (5.2)	5.0 (3.4–7.3)	<0.001
Peripheral arterial disease	25 (13.6)	125 (2.0)	7.9 (5.0–12.5)	<0.001	25 (13.8)	116 (3.1)	4.4 (3.1–7.9)	<0.001
ASCVD	87 (47.3)	528 (8.2)	10.0 (7.4–13.5)	<0.001	85 (47.0)	488 (13.1)	5.9 (4.3–8.0)	<0.001
Erectile dysfunction ^a, b^	57 (71.3)	447 (15.8)	13.2 (8.0–21.6)	<0.001	57 (73.1)	419 (25.0)	8.2 (4.9–13.6)	<0.001
Atrial fibrillation	84 (45.7)	166 (2.6)	31.6 (22.7–43.8)	<0.001	84 (46.4)	161 (4.3)	19.2 (13.8–26.8)	<0.001
Albuminuria	49 (26.6)	345 (5.4)	6.4 (4.5–9.0)	<0.001	49 (27.1)	284 (7.6)	4.5 (3.2–6.4)	<0.001
Low eGFR	88 (47.8)	436 (6.8)	12.5 (9.2–17.0)	<0.001	88 (48.6)	423 (11.3)	7.4 (5.4–10.1)	<0.001
Chronic kidney disease	102 (55.4)	654 (10.2)	10.9 (8.1–14.8)	<0.001	102 (56.4)	586 (15.7)	4.6 (3.5–6.0)	<0.001
Low cardiovascular risk	1 (0.5)	2144 (33.5)	0.01 (0.00–0.08)	<0.001	0 (0.0)	152 (4.1)	NE	NE
Moderate cardiovascular risk	7 (3.8)	1372 (21.4)	0.15 (0.07–0.31)	<0.001	7 (3.9)	1050 (28.1)	0.10 (0.05–0.22)	<0.001
High cardiovascular risk	18 (9.8)	1005 (15.7)	0.58 (0.36–0.95)	0.029	18 (9.9)	762 (20.4)	0.4 (0.3–0.7)	<0.001
Very high cardiovascular risk	158 (85.9)	1883 (29.4)	14.6 (9.6–22.2)	<0.001	156 (86.2)	1767 (47.4)	6.9 (4.5–10.6)	<0.001

^a^ N (males ≥18 years): 80 with HF; 2824 without HF. ^b^ N (males ≥50 years): 78 with HF; 1678 without HF. ASCVD: atherosclerotic cardiovascular disease; eGFR: estimated glomerular filtration rate; HDL-C: high-density lipoprotein cholesterol; NE: not estimable; WHtR: waist-to-height ratio. The definitions of the variables or clinical conditions are shown in Table S1 (Supplementary Materials).

**Table 4:** Clinical conditions and comorbidities independently associated with heart failure.

	Population ≥ 18 Years				Population ≥ 50 Years			
	Wald	β ^a^	OR Exp(β) ^b^	*p*-Value ^c^	Wald	β ^a^	OR Exp(β) ^b^	*p*-Value ^c^
Atrial fibrillation	179.9	2.54 (0.19)	12.66 (8.74–18.35)	<0.001	169.9	2.44 (0.19)	11.50 (8.00–16.61)	<0.001
Hypertension	39.9	1.70 (0.27)	5.48 (3.23–9.29)	<0.001	22.4	1.33 (0.28)	3.79 (2.18–6.57)	<0.001
Low eGFR	59.8	1.40 (0.18)	4.06 (2.84–5.78)	<0.001	52.5	1.30 (0.18)	3.66 (2.58–5.20)	<0.001
Coronary heart disease	29.0	1.15 (0.21)	3.17 (2.08–4.81)	<0.001	25.7	1.08 (0.21)	2.94 (1.94–4.45)	<0.001
Sedentary lifestyle	10.8	0.59 (0.18)	1.80 (1.27–2.55)	0.001	9.4	0.55 (0.18)	1.73 (1.22–2.46)	0.002
Stroke	7.1	0.66 (0.25)	1.92 (1.19–3.12)	0.008	5.4	0.57 (0.25)	1.76 (1.22–2.84)	0.021
Diabetes	5.5	0.43 (0.18)	1.54 (1.07–2.20)	0.020	4.3	0.38 (0.18)	1.46 (1.02–2.08)	0.037

^a^ β coefficient (± deviation). ^b^ Odds-ratio Exp (β) (95% confidence interval). ^c^ *p*-value of Wald test with one degree of freedom. eGFR: estimated glomerular filtration rate. The definitions of the variables or clinical conditions are shown in Table S1 (Supplementary Materials).

## Data Availability

Research data is available upon reasonable request.

## Supplementary Materials

The following supporting information can be downloaded at: https://www.mdpi.com/article/10.3390/jcm12154924/s1, Figure S1: Causes, comorbidities and complications of heart failure; Table S1: Variables or clinical conditions criteria.

## Abbreviations

AF	atrial fibrillation
ASCVD	atherosclerotic cardiovascular disease
CHD	coronary heart disease
CKD	chronic kidney disease
CVR	cardiovascular risk
CVRFs	cardiovascular risk factors
DM	diabetes mellitus
eGFR	estimated glomerular filtration rate
HF	heart failure
HTN	arterial hypertension
