# Supplementary material for: Heart Failure Prevalence Rates and Its Association with Other Cardiovascular Diseases and Chronic Kidney Disease: SIMETAP-HF Study
Source: J Clin Med. 2023 Jul 26;12(15):4924. doi: 10.3390/jcm12154924 (PMC10419820; doi:10.3390/jcm12154924)
Supplement: Supplementary file 1 [file jcm-12-04924-s001.zip › jcm-2522242-supplementary.pdf]

SUPPLEMENTARY MATERIALS

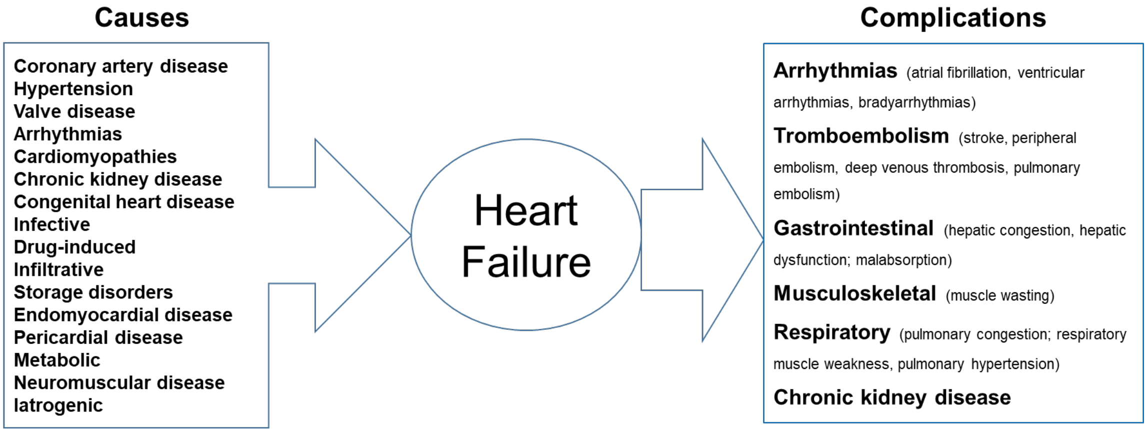

Figure S1. Causes, comorbidities and complications of heart failure. Modified of: McDonagh TA, et al., ESC Scientific Document Group. 2021 ESC Guidelines for the diagnosis and treatment of acute and chronic heart failure. Eur Heart J. 2021;42(36):3599–726. Coronary artery disease (myocardial infarction, angina, arrhythmias); Arrhythmias (atrial tachyarrhythmias, ventricular arrhythmias); Infective (viral myocarditis, Chagas disease, human immunodeficiency virus, Lyme disease); Drug-induced (anthracyclines, trastuzumab, VEGF inhibitors, immune checkpoint inhibitors, proteasome inhibitors, RAF+MEK inhibitors); Infiltrative (amyloid, sarcoidosis, neoplastic); Storage disorders (haemochromatosis, Fabry disease, glycogen storage diseases); Endomyocardial disease (radiotherapy, endomyocardial fibrosis/eosinophilia, carcinoid); Pericardial disease (calcification, infiltrative); Metabolic (endocrine disease, nutritional disease, autoimmune disease); Neuromuscular disease (Friedreich's ataxia, muscular dystrophy); Iatrogenic (postoperative fluid replacement or administration of steroids or non-steroidal anti-inflammatory drugs).

Table S1. Variables or clinical conditions criteria.

Clinical conditions or variables	Concepts and criteria
Current smoking	Any amount of tobacco use in the previous year.
Alcoholism	>21 standard drink units (SDU) of alcohol consumption per week (male), >14 SDU per week (female)<br>1 SDU is equivalent to 10 g of alcohol.
Sedentary lifestyle	Physical activity <150 minutes per week
Overweight (1)	Body mass index (BMI) 25.0–29.9 kg/m^2 (International Classification of Diseases, Tenth Revision, Clinical Modification [ICD-10-CM]: E66.3; International Classification of Primary Care, 2nd edition [ICPC-2]: T83)*
Obesity (1)	BMI ≥30 kg/m^2 (ICD-10-CM: E66.9; ICPC-2: T82)*
CUN-BAE-obesity (2)	Adiposity or body fat index CUN-BAE (*Clínica Universitaria de Navarra* - Body Adiposity Estimator):<br>• Male: −44.988 + (0.503 x age) + (3.172 x BMI) − (0.026 x BMI^2) − (0.02 x BMI x age) + (0.00021 x BMI^2 x age)<br>• Female: −44.988 + (0.503 x age) + 10.689 + (3.172 x BMI) − (0.026 x BMI^2) + (0.181 x BMI) − (0.02 x BMI x age) − (0.005 x BMI^2) + (0.00021 x BMI^2 x age)<br>Male >25%<br>Female >35%

Abdominal or central obesity (3)	Increased waist circumference (≥102 cm [male]; ≥88 cm [female]) determined with the subject standing using a flexible tape measure adjusted without compressing the skin, at the end of a normal expiration, locating the upper edge of the iliac crests and above that point surrounding the waist parallel to the floor.
High waist-to-height ratio (WHtR) (4)	Waist circumference/height ≥ 0.55
Arterial hypertension (HTN) (5)	Systolic blood pressure (SBP) ≥140 mmHg and/or diastolic blood pressure (DBP) ≥90 mmHg, using the average of ≥2 readings obtained on ≥2 occasions, or being on antihypertensive treatment (ICD-10-CM: I10, I15; ICPC-2: K86, K87)*
Diabetes mellitus (DM)	According to the American Diabetes Association (ADA) criteria (6): fasting plasma glucose (FPG) ≥126 mg/dL or glycated hemoglobin A1c (HbA1c) ≥6.5%, or plasma glucose ≥ 200 mg/dL at any time or with oral glucose tolerance test (ICD-10-CM: E10, E11; ICPC-2: T89, T90)*
Prediabetes	According to the ADA criteria (6): FPG between 100 and 125 mg/dL or HbA1c between 5.7% and 6.4% (ICD-10-CM: R73.09; ICPC-2: A91)*
Hypercholesterolaemia	Total cholesterol (TC) ≥200 mg/dL (ICD-10-CM: E78; ICPC-2: T93)*
Hypertriglyceridaemia	Triglycerides (TG) ≥150 mg/dL (≥1.7 mmol/L) (ICD-10-CM: E78; ICPC-2: T93)*
Low high-density lipoprotein cholesterol (HDL-C)	HDL-C <40 mg/dL (<1.03 mmol/L) (male)<br>HDL-C <50 mg/dL (<1.29 mmol/L) (female)
Non-high-density lipoprotein cholesterol (Non-HDL-C)	TC – HDL-C
Low-density lipoprotein cholesterol (LDL-C)	TC – HDL-C – (TG/5) in mg/dL (not valid for patients with TG >400 mg/dL)
Very low-density lipoproteins cholesterol and remnants (VLDL-C)	TC – HDL-C – LDL-C
Triglyceride-glucose index (TyG)	Ln (TG x FPG/2)
Atherogenic dyslipidaemia	Hypertriglyceridaemia and low HDL-C
Metabolic syndrome (MetS)	According to Harmonized Consensus of International Diabetes Federation task force on Epidemiology and Prevention, National Heart, Lung, and Blood Institute, American Heart Association, World Heart Federation, International Atherosclerosis Society, and International Association for the Study of Obesity (3):<br>At least, three of following factors for the European population:<br>• Increased waist circumference (≥102 cm [male]; ≥88 cm [female])<br>• FPG ≥100 mg/dL (≥5.6 mmol/L)<br>• TG ≥150 mg/dL (≥1.7 mmol/L)

	• HDL-C <40 mg/dL (<1.03 mmol/L) (males); <50 mg/dL (<1.29 mmol/L) (females)<br>• SBP ≥130 mmHg or DBP ≥85 mmHg or antihypertensive treatment
Hyperuricemia	Uric acid >7 mg/dL (male), >6 mg/dL (female) (ICD-10-CM: E79; ICPC-2: T92)*
Coronary heart disease (CHD)	Ischemic heart disease, acute myocardial infarction, acute coronary syndrome, coronary revascularization (ICD-10-CM: I20-I25; ICPC-2: K74, K75, K76)*
Cerebrovascular disease (stroke)	Cerebral ischemia, intracranial haemorrhage, transient ischemic attack (ICD-10-CM: I60-I66, I66, I67; ICPC-2: K89, K90K K91)*
Peripheral arterial disease (PAD)	Intermittent claudication, ankle-brachial index ≤ 0.9 (ICD-10-CM: I70.2, I73.9; ICPC-2: K92)*
Atherosclerotic vascular disease (ASCVD)	CHD, stroke, PAD (ICD-10-CM: I70)*
Heart failure (HF)	Record of HF diagnosis (ICD-10-CM: I50; ICPC-2: K77)* in the patient's medical record, without differentiating by phenotype based on measurement of left ventricular ejection fraction or based on severity of symptoms and physical activity.
Atrial fibrillation (AF)	Record of AF diagnosis (ICD-10-CM: I48; ICPC-2: K78)* in the patient's medical record.
Estimated glomerular filtration rate (eGFR)	According to Chronic Kidney Disease Epidemiology Collaboration (CKD-EPI) equations (7):<br>Women with creatinine ≤ 0.7 mg/dL= 144 x $(\text{creatinine})^{-0.329}$ x $(0.993)^{\text{age}}$ mL/min/1.73 m^2<br>Women with creatinine > 0.7 mg/dL= 144 x $(\text{creatinine})^{-1.209}$ x $(0.993)^{\text{age}}$ mL/min/1.73 m^2<br>Men with creatinine ≤ 0,9 mg/dL= 141 x $(\text{creatinine})^{-0,411}$ x $(0,993)^{\text{age}}$ mL/min/1.73 m^2<br>Men with creatinine > 0,9 mg/dL= 141 x $(\text{creatinine})^{-1,209}$ x $(0,993)^{\text{age}}$ mL/min/1.73 m^2
Low eGFR (8)	eGFR <60 mL/min/1.73 m^2 according to CKD-EPI (7)
Albuminuria (8)	Urine albumin-creatinine ratio (ACR) ≥30 mg/g (including proteinuria [ACR >300 mg/g] (ICD-10-CM: R80; ICPC-2: U98)*
Chronic kidney disease CKD (8)	Low eGFR and/or albuminuria (ICD-10-CM: N18; ICPC-2: U99)*
Cardiovascular risk (CVR) categories	Low, moderate, high and very high CVR according to SCORE *(systematic coronary risk evaluation)* (9) or SCORE-OP (10) for low-risk countries (9)

* National Center for Health Statistics (NCHS). International Classification of Diseases, Tenth Revision, Clinical Modification (ICD-10-CM). [Accessed 15 June, 2023]. Available from: https://www.cdc.gov/nchs/icd/icd-10-cm.htm#print. * World Health Organization. (2009). International Classification of Primary Care, -ICPC-2. [Accessed 15 June, 2023]. Available from: https://www.who.int/standards/classifications/other-classifications/international-classification-of-primary-care.

1. WHO Consultation on Obesity (1999: Geneva, Switzerland) & World Health Organization. (2000). Obesity: preventing and managing the global epidemic: Report of a WHO consultation. WHO technical report series; 894. [Accessed 15 June, 2023]. Available from: https://apps.who.int/iris/handle/10665/42330.
2. Gómez-Ambrosi J, Silva C, Catalán V, Rodríguez A, Galofré JC, Escalada J, et al. Clinical usefulness of a new equation for estimating body fat. Diabetes Care. 2012;35:383–8, https://doi.org//10.2337/dc11-1334.
3. Alberti KGMM, Eckel RH, Grundy SM, Zimmet PZ, Cleeman JI, Donato KA, et al. Harmonizing the metabolic syndrome: A joint interim statement of the International Diabetes Federation task force on Epidemiology and Prevention; National Heart, Lung, and Blood Institute; American Heart Association; World Heart Federation; International Atherosclerosis Society; and International Association for the Study of Obesity. Circulation. 2009;120:1640–5, https://doi.org/10.1161/CIRCULATIONAHA.109.192644.
4. Romero-Saldaña M, Fuentes-Jiménez FJ, Vaquero-Abellán M, Álvarez-Fernández C, Aguilera-López MD, Molina-Recio G. Predictive capacity and cutoff value of waist-to-height ratio in the incidence of metabolic syndrome. Clin Nurs Res. 2019;28:676–91, https://doi.org/10.1177/1054773817740533.
5. Williams B, Mancia G, Spiering W, Agabiti Rosei E, Azizi M, Burnier M, et al. 2018 ESC/ESH Guidelines for the management of arterial hypertension of the European Society of Cardiology (ESC) and the European Society of Hypertension (ESH). The Task Force for the management of arterial hypertension of the European Society of Cardiology (ESC) and the European Society of Hypertension (ESH). Eur Heart J. 2018;39:3021–104, https://doi.org/10.1093/eurheartj/ehy339.
6. ElSayed NA, Aleppo G, Aroda VR, Bannuru RR, Brown FM, Bruemmer D, et al.; American Diabetes Association. 2. Classification and Diagnosis of Diabetes: Standards of Care in Diabetes-2023. Diabetes Care. 2023;46(Suppl 1): S19-S40, https://doi.org/10.2337/dc23-S002.
7. Levey AS, Stevens LA, Schmid CH, Zhang YL, Castro AF 3rd, Feldman HI, et al., CKD-EPI (Chronic Kidney Disease Epidemiology Collaboration). A new equation to estimate glomerular filtration rate. Ann Intern Med. 2009;150:604–12, https://doi.org/10.7326/0003-4819-150-9-200905050-00006.
8. Kidney Disease: Improving Global Outcomes (KDIGO) CKD Work Group. KDIGO 2012 Clinical practice guideline for the evaluation and management of chronic kidney disease. Kidney Int Suppl. 2013;3:5–14, https://doi.org/10.1038/kisup.2012.77.
9. Piepoli MF, Hoes AW, Agewall S, Albus C, Brotons C, Catapano AL, et al. 2016 European Guidelines on cardiovascular disease prevention in clinical practice. The Sixth Joint Task Force of the European Society of Cardiology and Other Societies on Cardiovascular Disease Prevention in Clinical Practice (constituted by representatives of 10 societies and by invited experts). Developed with the special contribution of the European Association for Cardiovascular Prevention & Rehabilitation (EACPR). Eur Heart J. 2016;37:2315–81, https://doi.org//10.1093/eurheartj/ehw106.
10. Cooney MT, Selmer R, Lindman A, Tverdal A, Menotti A, Thomsen T, et al. Cardiovascular disease risk estimation in older persons: SCORE O.P. Eur J Prev Cardiol. 2016;23:1093–103, https://doi.org/10.1177/2047487315588390.
